# Impact of the Siewert Classification on the Outcome of Patients Treated by Preoperative Chemoradiotherapy for a Nonmetastatic Adenocarcinoma of the Oesophagogastric Junction

**DOI:** 10.1155/2015/404203

**Published:** 2015-09-10

**Authors:** Laurence Moureau-Zabotto, Eric Teissier, Didier Cowen, David Azria, Steve Ellis, Michel Resbeut

**Affiliations:** ^1^Department of Radiation Therapy, Institut Paoli Calmettes, 232 boulevard de Sainte Marguerite, 13009 Marseille, France; ^2^Azurean Cancer Center, Mougins, France; ^3^Department of Radiation Therapy, Timone Academic Hospital and North Academic Hospital, Marseille, France; ^4^Department of Radiation Therapy, Val d'Aurelle Cancer Center, Montpellier, France; ^5^Catalan Oncology Center, Perpignan, France; ^6^French Red Cross Center, Toulon, France

## Abstract

The aim of the study is to analyze the impact of the Siewert classification on the pathological complete response (pcR), pattern of failure, and general outcome of patients treated, by preoperative chemoradiotherapy and surgery for an gastroesophageal junction adenocarcinoma (OGJA). From 2000 to 2008, the charts of 68 patients were retrospectively reviewed. Tumor staging reported was UST1/T2/T3/T4/unknown, respectively, *n* = 1/7/54/5/1 patients, and N0/N1/unknown, respectively, *n* = 9/58/1 patients. Patients received primary external-beam radiotherapy with concurrent chemotherapy followed by surgical resection (Siewert I: upper oesogastrectomy; Siewert II/III: total gastrectomy with lower oesophagectomy). Overall survival (OS), overall relapse rate (ORR), cumulative rate of local (CRLR), nodal (CRNR), and metastatic (CRMR) relapse, and their prognostic factors were retrospectively analyzed. Median follow-up was 77.5 months. Median OS was 41.7 ± 5.2 months. The 3-year ORR was 48%. Using univariate analysis ORR was significantly increased for patients with Siewert II/III compared to Siewert I tumors (27.3% versus 62%, *p* = 0.047). Siewert I tumors had also statistically lower CRNR and CRMR compared to Siewert II/III tumors (0/9.1% versus 41.3/60.2% resp., *p* = 0.012), despite an equivalent cumulative rate of local relapse and pathological complete response rate between the three groups. For OGJA treated with preoperative CRT and surgery, ORR and CRMR were lower for patients with Siewert I tumors in comparison with Siewert II/III tumors.

## 1. Background

Oesophageal and gastroesophageal junction cancer is the eighth most common form of cancer, with almost 482000 new cases and 407000 deaths in the world in 2008 [1]. The incidence of adenocarcinoma has increased in compaison to all other histology [2, 3]. Although early-stage localized disease with no evidence of nodal spread and with invasion confined to the submucosa has a good prognosis after complete local resection, more advanced lesions benefit from combined modality therapy with more extensive resection to ensure negative margins, including lymph node dissection. After primary surgery 25% of patients had microscopically positive resection margins, and the 5-year survival rate remained poor, generally under 40% [4–6].

Recently, the seventh edition of the TNM classification improved the predictive ability for cancers of the oesophagus. Differences in patient characteristics, pathogenesis, and especially survival clearly identify adenocarcinomas and squamous cell carcinoma of the oesophagus as 2 separate tumor entities requiring differentiated therapeutic concepts [7]. Meta-analysis suggests an evident survival benefit for preoperative chemoradiotherapy and, to a lesser extent, for chemotherapy in patients with adenocarcinoma of the oesophagus. In this meta-analysis, preoperative radiochemotherapy increased survival whatever the histological subtypes, whereas the benefit in survival for neoadjuvant chemotherapy was observed only for adenocarcinomas [8, 9].

Siewert recognized the need for a tumor classification based on anatomic location and proposed a classification scheme for distal esophageal and gastroesophageal junction neoplasms to guide therapy and allow for a more meaningful study [10, 11]. The classification of adenocarcinomas of the gastroesophageal junction (AEG) in three types, AEG type I, type II, and type III, shows marked differences between the tumor entities and is recommended for selection of a proper surgical approach. By extrapolation, this classification has been extensively used to adapt the preoperative treatment: patients presenting with a Siewert type 1 tumor are generally considered and treated as patients with oesophagus cancer and receive, for operable locally advanced tumors, concomitant radiation chemotherapy followed by surgery, whereas patients presenting with Siewert type 2 and 3 tumors are generally considered and treated as patients with gastric cancers by perioperative chemotherapy [12]. However, there is no rational basis supported by evidence in the literature to explain such a difference in current preoperative practices according to the Siewert classification.

In this retrospective study, we analyzed the outcome of a series of 68 patients treated, independently of the Siewert classification of their tumor, by concomitant preoperative radiochemotherapy followed by surgery, and the impact of the Siewert classification on the pathological response, pattern of failure, and general outcome of the patients.

## 2. Methods

### 2.1. Patient's Selection

Between January 2000 and December 2008, 159 patients were treated by radiotherapy with or without +/− concomitant chemotherapy for a nonmetastatic adenocarcinoma of the gastroesophageal junction in four French cancer centers. During this period and before the publication of the MAGIC Trial and the French FFCD Trial results [13, 14], all patients requiring neoadjuvant treatment for a nonmetastatic adenocarcinoma of the gastroesophageal junction were treated by neoadjuvant radiotherapy with or without +/− concomitant chemotherapy in these cancer centers. Patients presenting with distant metastasis and/or a squamous cell carcinoma were excluded. Among these 159 patients, the charts of 68 patients who received preoperative radiotherapy with concomitant chemotherapy, followed by surgery, were selected for this study. This analysis was approved by local institutional review boards.

All patients underwent an oeso-gastroscopy, an gastroesophageal endoscopic ultrasound (EUS), and a computed thoracoabdominal and pelvic tomography (CT) scan. Positron emission tomography was not used routinely. All the tumors were restaged on the basis of the seventh edition of the American Joint Committee on Cancer tumor staging criteria [15]. Laparoscopy was never performed as a diagnostic procedure. T and N stage were assessed by EUS and CT scan. Lymph nodes measuring more than 1 cm on the CT scan were considered to be clinically involved. Tumors were also classified using the Siewert classification [10, 11]: Siewert 1 tumors were located only in the lower esophagus, Siewert 2 tumors were located in both the lower esophagus and the upper gastric wall, and Siewert 3 tumors concerned only the gastric part of the gastroesophageal junction, without involvement of the esophagus.

### 2.2. Treatments

Irradiation consisted of external-beam radiotherapy delivering a mean total dose of 44.5 Gy (range, 36–50 Gy), at 1.8 to 2 Gy per fraction, with a 3- or 4-field technique. The irradiated volume took into account the tumor size and the risk of lymph node involvement. Irradiation modalities were set at the discretion of each attending radiation oncologist. Two patients received a boost in the gross tumor volume delivering 10 and 14.4 Gy, respectively. Mean total treatment time for radiotherapy was 36.5 days (24–60). The dose was prescribed to the International Commission on Radiation Units and Measurements point and delivered through linear accelerators by use of high-energy photons (≥10 MV). During the radiation therapy, all patients received concurrent chemotherapy, mainly based on cisplatin and 5FU regimen (*n* = 26, 38.3%), cisplatin or carboplatin alone (*n* = 39, 57.3%), or FOLFOX (*n* = 3, 4.4%). Six patients (8.8%) previously received 2 additional cycles of neoadjuvant chemotherapy based on cisplatin and 5FU regimen. Seven (10, 3%) patients received adjuvant chemotherapy based on FOLFIRI (*n* = 4, 5.9%) or cisplatin plus 5FU (*n* = 3, 4, 4%).

After a mean delay of 58.8 days (26–182), patients underwent surgery, which was represented by an oeso-gastrectomy polar superior (Lewis Santy procedure) for Siewert 1 tumors, whereas patients with Siewert 2 or 3 tumors underwent an extended total gastrectomy with resection of the lower esophagus, associated with a D2-lymphadenectomy.

Patients were seen for the first follow-up 8 to 12 weeks after the end of the treatment and thereafter every 3 or 4 months up to 2 years. Afterwards, follow-up was planned every 6 months, until progression or death. Follow-up encompassed a clinical examination and a blood sample including tumor markers, as well as a CT scan. Endoscopic control was generally carried out every year.

### 2.3. Statistics

The follow-up was calculated from the date of diagnosis to the date of last follow-up, using the Schemper method [16]. The differences between each group were evaluated by use of the chi2 test or the Fisher exact test for categorical variables and the Mann-Whitney *U* test for continuous variables. Survival rates were estimated by use of the Kaplan-Meier method [17]. The overall relapse rate (ORR) was defined by the date of diagnosis and the date of the first relapse whatever the site of relapse. Cumulative rate of local recurrence (CRLR) was defined by the date of diagnosis and the date of the first local relapse, cumulative rate of nodal recurrence (CRNR) was defined by the date of diagnosis and the date of the first nodal relapse, and the cumulative rate of metastatic recurrence (CRMR) was defined by the date of the diagnosis and the date of the first distant relapse. Overall survival (OS) was defined by the interval between the date of the diagnosis and the date of death or last follow-up. The progression-free survival (PFS) was defined between the date of diagnosis and the date of the first relapse and/or death. Univariate and multivariate prognostic analyses were performed with the Cox regression model [18]. Variables associated with survival with *p* < 0.2 in the univariate analysis were included in the multivariate regression. The variables included in the model were age as continuous variable and age (<62 versus ≥62 years, i.e., median age), gender, T and N stage, tumor length, the Siewert classification, and overall treatment time (OTT). All the variables included in the models were categorical. Analyses were performed with SPSS statistical software, version 16.0 (SPSS, Chicago, IL). All statistical tests were 2-sided, and *p* ≤ 0.05 indicated statistical significance.

## 3. Results

The characteristics of the patients are reported in Table 1. The median age of patients was 61 years (26–79), and sex ratio was F/M: 8/60.  Table 2 summarizes the correlation between the patient's characteristics and the Siewert classification of tumors.

### 3.1. Pathological Complete Response

Pathological complete response was observed in 16 patients (23.5%). No statistical difference was observed in terms of pathological complete tumor between the three Siewert groups of patients (Table 3).

### 3.2. Overall Survival and Cause of Deaths

**Figure 1:**
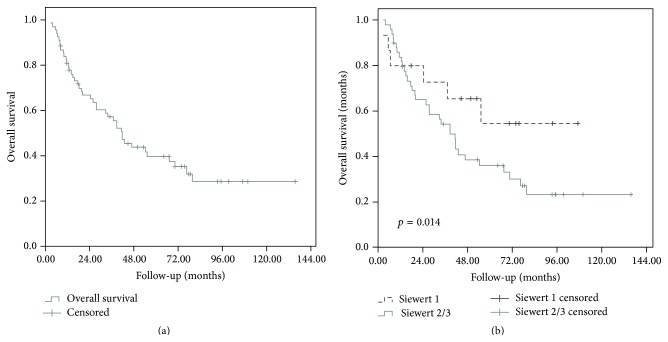
Overall survival for overall population (a) and according to the Siewert classification (b).

Median follow-up was 77.5 months (range, 3–135 months). At the end of follow-up, 42 (61.8%) patients had died: 30 (44.1%) died of disease evolution, 7 (10.3%) patients died of treatment related complications including 5 (7.3%) patients who died in the two months following surgery, 3 patients (4.4%) died of other causes, and for 2 (2.9%) patients the cause of death was unknown. The 1- and 3-year OS rates were 80.8% and 57.2%, respectively, with a median of 41.7 ± 5.2 months (Figure 1(a)). Using univariate analysis, age as a continuous variable (*p* < 0.0001), tumor size as a continuous variable (*p* < 0.0001), Karnofsky performance status (*p* = 0.05), and ypN (*p* < 0.0001) classification significantly influenced OS. Using multivariate analysis, ypN status was the only factor independently influencing overall survival (*p* = 0.001). Results of uni- and multivariate analysis for OS are detailed in Table 4(a).

Overall survival according to the Siewert classification was summarized in Table 3: OS was not statistically different between the 3 different Siewert groups (Figure 1(b)).

### 3.3. Progression-Free Survival and Pattern of Failure

At the end of follow-up, 32 (47.1%) patients presented a relapse: 15 (22.1%) presented a local relapse, 13 (19.1%) presented a nodal relapse, 27 (39.7%) presented a distant relapse, and among them, 10/27 presented both local and distant relapse. The 1- and 3-year PFS rates were 68.7% and 41.6%, respectively, with a median of 25.6 ± 2.6%. The 1- and 3-year overall relapse rates were 24.8% and 48%, respectively. The 1- and 3-year CRLR rates were 6.7% and 24.3%, respectively. The 1- and 3-year CRMR rate were 22.2% and 40.6%, respectively.

**Figure 2:**
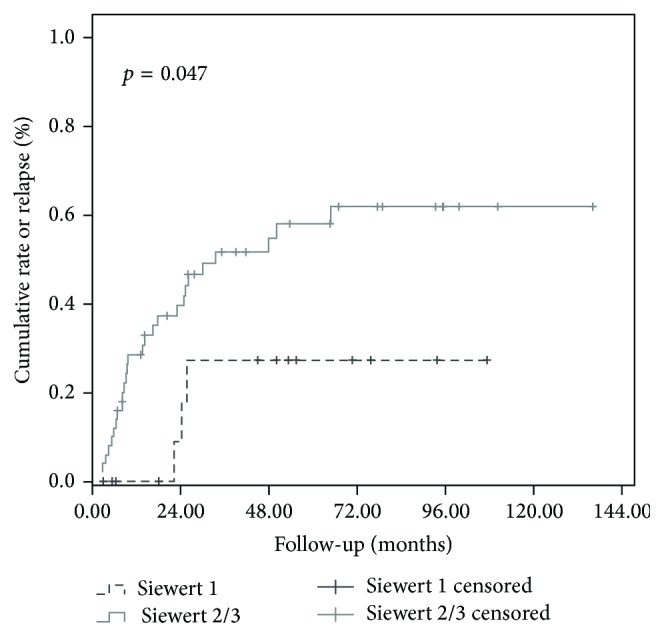
Overall recurrence rate according to the Siewert classification (Siewert type 1 versus Siewert types 2 and 3, *p* = 0.047).

Using univariate analysis, age as a continuous variable (*p* < 0.0001), age (≤62 versus >62 years) (*p* = 0.04), tumor size as a continuous factor (*p* < 0.0001), Siewert classification (Siewert 1 versus Siewert 2/3, *p* = 0.047, Figure 2), and ypN (*p* < 0.003) significantly influenced ORR. Using multivariate analysis, age (*p* = 0.02) and ypN status (0.006) independently influenced ORR. Results of uni- and multivariate analysis for ORR are detailed in Table 4(b).

### 3.4. Mean ORR, CRLR, CRNR, and CRMR according to the Siewert Classification Were Detailed in Table 3

**Figure 3:**
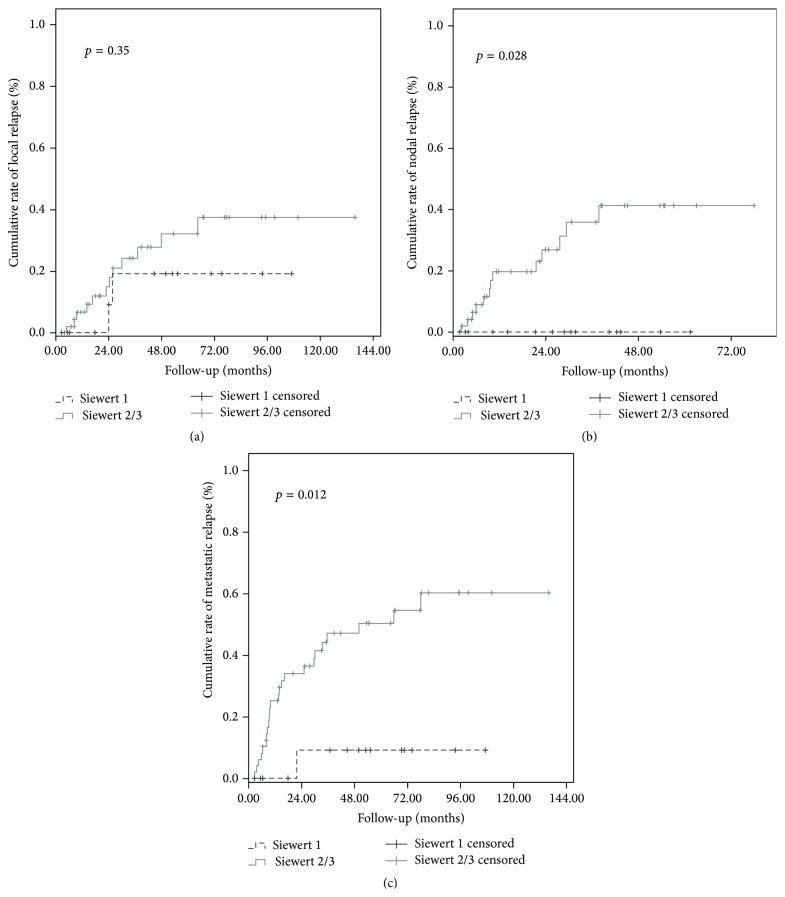
Cumulative rate of local recurrence (CRLR) (a), nodal recurrence (NRLR) (b), and metastatic recurrence (MRLR) (c) according to the Siewert classification (Siewert type 1 versus Siewert types 2 and 3).

ORR was statistically lower for Siewert 1 tumors in comparison to Siewert 2 and 3 tumors (*p* = 0.047, Figure 2). There was no statistical difference in terms of CRLR between the three Siewert groups; however, Siewert 1 tumors had statistically lower CRNR and CRMR in comparison to Siewert 2 and 3 tumors (Table 3, Figures 3(a), 3(b), and 3(c), resp.)

## 4. Discussion

This study is, to our knowledge, the first to focus on the impact of the Siewert classification on the pathological response, on the pattern of failure, and also on the general outcome of the patients after concomitant preoperative chemoradiation. This study concerned a consequent number of patients, treated with preoperative radiochemotherapy, avoiding misleading factors such as nonhomogeneous population in terms of patient clinical staging and therapeutic strategy. With regard to study limitations, this analysis was conducted in a retrospective, multicentric, and nonrandomized manner.

Pathological response rate observed in this study was 16/68 (22%), in the range to the rate already published in the literature [5, 19, 20]. In our study, no statistical difference was observed in terms of pathological complete tumor response between Siewert 1, 2, and 3 groups of patients. This could suggest that these three groups have the same radiosensitivity, which could have its importance, as it is well described that pathological complete response after neoadjuvant radiotherapy is an independent prognostic factor of overall survival [20]. Based on a large cohort, Wang et al. have demonstrated that over a recent 10-year period, the three subtypes of adenocarcinoma of the gastroesophageal junction showed different histological changing trends, suggesting heterogeneous characteristics of the three Siewert types of adenocarcinoma of the gastroesophageal junction. However nothing was published concerning the difference in terms of radiosensitivity of these three subtypes, and this specific point could be evaluated in a future clinical trial [21].

In our study the overall and progression-free survival rates are in the range of those already reported in the literature [5, 6, 19]. OS was not statistically different between the 3 different Siewert groups, whereas we observed a lower overall relapse rate in patients treated for Siewert 1 tumors in comparison to Siewert 2 and 3 tumors, with also a lower rate of nodal and metastatic relapse for this specific group. This, however, only held on univariate analysis and significance was lost in multivariate analysis. In this study, the number of patients with pathological nodal involvement has a trend to be more favorable in Siewert 1 group (11 ypN0, 4 ypN+) in comparison with Siewert type 2/3 groups (21 ypN0, 24 ypN+), which could also explain the best results observed in this study for Siewert type 1 group but this difference does not reach statistical significance (*p* = 0.07), and when we look at the pretreatment classification, there is no difference in terms of USN classification between the two Siewert groups (*p* = 0.3).

The other prognostic factors found significant in multivariate analysis for ORR in this study were in concordance with other published data in particular age [22], tumor size [5], and nodal status [23].

Xiao et al. have studied the difference in the characteristics of lymphatic metastasis and its impact in terms of surgical approach in a retrospective series of 228 patients, showing that for type I the pattern of lymph node metastasis is similar to that of the distal esophageal carcinoma (with a rate of 44%), for type II they observed a high rate of lymph node metastasis 66.9% (1/3 in the thoracic cage and 2/3 in the abdomen), and for type III, the rate of lymph node metastasis was also 66.9% including mainly abdominal metastasis (70.4%) [24]. The high rate of nodal involvement observed in Siewert types 2 and 3 is in accordance with our statistical difference in terms of nodal and metastasis rate and suggests that preoperative concomitant chemoradiotherapy is not efficient enough to control microscopic distant invasion with further significant uncertainties concerning the clinical target volume. Two other recent publications focused on the same topic, though being in nonirradiated patients. Reeh et al. have described type II OGJA as a more aggressive tumour with higher recurrence rates in comparison with type I OGJA, suggesting a potential benefit from more aggressive surgical treatment for such tumors [25]. More recently, Curtis et al. published the results of a large prospective study including patients who received preoperative chemotherapy instead of CRT and described type III tumours as larger and associated with frequent histological perineural and vascular invasion, although this did not translate into more lymph node metastasis [26]. This hypothesis suggests that Siewert 2 and 3 tumors represent biologically different tumors and could need the use of more aggressive treatment in particular the use of more efficient and aggressive multimodalities treatments. This is also concordant with the publication of Hasegawa et al. where Siewert type 2/3 tumors are more considered as gastric tumors than as esophagus tumors [12] and could be treated as gastric cancer by perioperative chemotherapy, with or without preoperative chemoradiotherapy as suggested by the results of a recent phase II randomized trial [19].

## 5. Conclusions

For gastroesophageal adenocarcinomas, treated with concomitant preoperative chemoradiotherapy, our study pointed out a difference in terms of ORR between Siewert 1 and 2/3 tumors, with an increasing rate of distant relapse for Siewert 2 and 3 tumors in comparison with Siewert I tumors, despite an equivalent local control rate and pathological response rate between these three groups. This paper also found well-described prognostic factors to be significant in particular nodal status, in accordance with previous published data, and further study is needed to determine whether there is a real difference between Siewert group 1 and Siewert 2/3 patients, as the reliability of the Siewert classification is actually controverted [27].

## Figures and Tables

**Table 1:** Patient, tumor, and characteristics.

	Patients
	*N* = 68
Center of treatment, *n* (%)	
1	6 (8.8)
2	56 (82.4)
3	1 (1.5)
4	5 (7.4)
Mean age (years, range)	61 (26–79)
Gender (*n*, %)	
Men	60 (88.2)
Women	8 (11.8)
Karnofsky performance status (range)	
Median	90 (50–100)
UST stage (*n*, %)	
UST1	1 (1.5)
UST2	7 (10.3)
UST3	54 (79.4)
UST4	5 (7.4)
NA	1 (1.5)
USN stage (*n*, %)	
USN0	9 (13.2)
USN1	58 (85.3)
NA	1 (1.5)
Mean tumoral length (mm, range)	48.2 mm (19–110)
Siewert classification, *n* (%)	
1	15 (22.1)
2	45 (66.2)
3	5 (7.4)
NA	3 (4.4)
ypT, *n* (%)	
ypT0	16 (23.5)
ypT1	6 (8.8)
ypT2	18 (26.5)
ypT3	18 (26.5)
ypT4	3 (4.4)
NA	7 (10.3)
ypN, *n* (%)	
ypN0	33 (48.5)
ypN1	27 (39.7)
ypN2	3 (4.4)
NA	5 (7.4)
Histological tumoral size (mm, range)	22.4 (0–70)

NA: nonavailable.

**Table 2:** Correlation between patients characteristics and Siewert classification.

	Siewert 1	Siewert 2 + 3	*p* (spearman correlation)
Cancer center, *n* (%)			
1	1 (16.7)	5 (83.3)	0.9
2	13 (24.1)	41 (75.9)	
3	0 (0)	1 (100)	
4	1 (25)	3 (75)	
Karnofsky index, *n* (%)			
80–100	13 (25)	39 (75)	0.5
<80	1 (14.3)	6 (85.7)	
Sex, *n* (%)			
Women	0	6 (100)	0.2
Men	15 (25.4)	44 (74.6)	
Age, *n* (%)			
≤62 years	6 (17.6)	28 (82.4)	0.2
>62 years	9 (29)	22 (71)	
Histologic differentiation, *n* (%)			
Well differentiated	3 (30)	7 (70)	0.3
Intermediate	4 (44.4)	5 (55.6)	
Undifferentiated	3 (17.6)	14 (82.4)	
UST, *n* (%)			
UST 1-2	4 (57.1)	3 (42.9)	0.06
UST 3-4	11 (19.3)	46 (80.7)	
USN, *n* (%)			
USN 0	1 (11.1)	8 (88.9)	0.3
USN 1	14 (25.5)	41 (74.5)	
Tumoral length, *n* (%)			
<50 mm	8 (32)	17 (68)	0.3
≥50 mm	7 (23.3)	23 (76.7)	
ypT, *n* (%)			
pT 0–2	12 (30)	28 (70)	0.2
pT 3-4	3 (16.7)	15 (83.3)	
ypN, *n* (%)			
pN0	11 (34.4)	21 (65.6)	0.07
pN ≥ 1	4 (14.3)	24 (85.7)	

**Table 3:** Outcome of patients according to Siewert classification.

Outcome	Siewert 1	Siewert 2	Siewert 3	Siewert 2 + 3	Siewert 1 versus 2 versus 3 *p*	Siewert 1 versus (2 + 3) *p*
Mean						
OS ± SE (months)	70 ± 11.7	53.2 ± 7.2	61.3 ± 18.8	56.7 ± 7.2	0.18	0.14
Mean						
ORR ± SE (%)	27.3 ± 13.4	76.5 ± 8.3	20 ± 17.9	62 ± 7.9	0.052	0.047
Mean						
CRLR ± SE (%)	19.2 ± 10.1	41.8 ± 10.1	0	37.5 ± 9.2	0.27	0.35
Mean						
CRNR ± SE (%)	0	45.1 ± 10.7	20 ± 17.9	41.3 ± 9.7	0.08	0.028
Mean						
CRMR ± SE (%)	9.1 ± 8.7	65.2 ± 9.7	75 ± 21.7	60.2 ± 8.9	0.017	0.012
pCR, *n* (%)						
Yes	6 (40)	8 (21.1)	2 (40)	10 (23.3)	0.31	0.21
No	9 (60)	30 (78.9)	3 (60)	33 (76.7)		
NA (*n* = 10)						
Cause of death, *n* (%)						
Disease progression	2 (33.3)	25 (78.1)	1 (50)	26 (76.5)	0.09	0.06
Complication	3 (50)	3 (9.4)	1 (50)	4 (11.8)		
Other causes	1 (16.7)	4 (12.5)	0	4 (11.8)		
Cause of death, *n* (%)						
Disease progression	2 (33.3)	25 (78.1)	1 (50)	26 (76.5)	0.07	0.03
Other causes	4 (66.7)	7 (21.9)	1 (50)	8 (23.5)		

OS: overall survival; ORR: overall relapse rate; SE: standard error; CRLR: cumulative rate of local relapse; CRNR: cumulative rate of nodal relapse; CRMR: cumulative rate of metastatic relapse; pCR: pathological complete response; NA: nonavailable.

**(a):** 

	Univariate analysis			Multivariate analysis	
	Alive (%)		*p* ^(1)^	HR [CI 95%]^(2)^	*p* ^(3)^
	No	Yes			
Total (*n* = 68)	42 (62)	26 (38)	—	—	—
pT					
ypT0/T1/T2	22 (55%)	18 (45%)	0.073	1	0.6
ypT3/T4	16 (76%)	5 (24%)		1.2 [0.5–2.7]	
pN					
ypN0	13 (39%)	20 (61%)	<0.0001	1	—
ypN0+	26 (87%)	4 (13%)		4.1 [1.83–9.2]	0.001
Siewert classification					
Siewert 1	6 (40%)	9 (60%)	0.14	1	—
Siewert 2/3	34 (68%)	16 (32%)		1.5 [0.6–3.7]	0.4
Performance status					
PS 0-1	20 (38%)	33 (62%)	0.05	1	0.7
PS 2-3	2 (25%)	6 (75%)		1.2 [0.4–3.9]	

**(b):** 

	Univariate analysis			Multivariate analysis	
	Relapse (%)		*p* ^(1)^	HR (CI 95%)^(2)^	*p* ^(3)^
	No	Yes			
Total	36 (53)	32 (47)	—	—	—
ypT					
ypT0/T1/T2	24 (60%)	16 (40%)	0.13	1	—
ypT3/T4	73 (90%)	8 (10%)		1.2 [0.5–2.8]	0.6
ypN					
ypN0	23 (70%)	10 (30%)	0.003	1	—
ypN0+	12 (40%)	18 (60%)		3.4 [1.4–8.1]	0.005
Age (years)					
≤62	14 (39%)	22 (61%)	0.038	1	—
>62	22 (69%)	10 (31%)		0.36 [0.15–0.86]	0.02
Siewert classification					
Siewert 1	12 (80%)	3 (20%)	0.047	1	—
Siewert 2/3	23 (46%)	27 (54%)		2.8 [0.8–9.5]	0.1

^(1)^“*p* value” of Log-rank test.

^(2)^Hazard-Ratio (95% confidence interval).

^(3)^“*p* value” of Cox model.

## References

[1] Ferlay J., Shin H.-R., Bray F., Forman D., Mathers C., Parkin D. M. (2010). Estimates of worldwide burden of cancer in 2008: GLOBOCAN 2008. *International Journal of Cancer*.

[2] Brown L. M., Devesa S. S., Chow W.-H. (2008). Incidence of adenocarcinoma of the esophagus among white Americans by sex, stage, and age. *Journal of the National Cancer Institute*.

[3] Pohl H., Welch H. G. (2005). The role of overdiagnosis and reclassification in the marked increase of esophageal adenocarcinoma incidence. *Journal of the National Cancer Institute*.

[4] Kelsen D. P., Ginsberg R., Pajak T. F. (1998). Chemotherapy followed by surgery compared with surgery alone for localized esophageal cancer. *The New England Journal of Medicine*.

[5] Stahl M., Walz M. K., Stuschke M. (2009). Phase III comparison of preoperative chemotherapy compared with chemoradiotherapy in patients with locally advanced adenocarcinoma of the esophagogastric junction. *Journal of Clinical Oncology*.

[6] Van Hagen P., Hulshof M. C. C. M., van Lanschot J. J. B. (2012). Preoperative chemoradiotherapy for esophageal or junctional cancer. *The New England Journal of Medicine*.

[7] Gertler R., Stein H. J., Langer R. (2011). Long-term outcome of 2920 patients with cancers of the esophagus and esophagogastric junction: evaluation of the new union internationale contre le cancer/American joint cancer committee staging system. *Annals of Surgery*.

[8] Gebski V., Burmeister B., Smithers B. M., Foo K., Zalcberg J., Simes J. (2007). Survival benefits from neoadjuvant chemoradiotherapy or chemotherapy in oesophageal carcinoma: a meta-analysis. *Lancet Oncology*.

[9] Sjoquist K. M., Burmeister B. H., Smithers B. M. (2011). Survival after neoadjuvant chemotherapy or chemoradiotherapy for resectable oesophageal carcinoma: an updated meta-analysis. *The Lancet Oncology*.

[10] Siewert J. R., Holscher A. H., Becker K., Gossner W. (1987). Cardia cancer: attempt at a therapeutically relevant classification. *Chirurg*.

[11] Siewert J. R., Stein H. J. (1998). Classification of adenocarcinoma of the oesophagogastric junction. *British Journal of Surgery*.

[12] Hasegawa S., Yoshikawa T., Aoyama T. (2013). Esophagus or stomach? The seventh TNM classification for siewert type II/III junctional adenocarcinoma. *Annals of Surgical Oncology*.

[13] Cunningham D., Allum W. H., Stenning S. P. (2006). Perioperative chemotherapy versus surgery alone for resectable gastroesophageal cancer. *The New England Journal of Medicine*.

[14] Ychou M., Boige V., Pignon J.-P. (2011). Perioperative chemotherapy compared with surgery alone for resectable gastroesophageal adenocarcinoma: an FNCLCC and FFCD multicenter phase III trial. *Journal of Clinical Oncology*.

[15] Edge S. E., Byrd D. R., Compton C. C. (2009).

[16] Schemper M., Smith T. L. (1996). A note on quantifying follow-up in studies of failure time. *Controlled Clinical Trials*.

[17] Kaplan E. L., Meier P. (1958). Nonparametric estimation from incomplete observations. *Journal of the American Statistical Association*.

[18] Cox D. R. (1972). Regression models and life-tables. *Journal of the Royal Statistical Society, Series B: Methodological*.

[19] Burmeister B. H., Thomas J. M., Burmeister E. A. (2011). Is concurrent radiation therapy required in patients receiving preoperative chemotherapy for adenocarcinoma of the oesophagus? A randomised phase II trial. *European Journal of Cancer*.

[20] Van Hagen P., Wijnhoven B. P. L., Nafteux P. (2013). Recurrence pattern in patients with a pathologically complete response after neoadjuvant chemoradiotherapy and surgery for oesophageal cancer. *British Journal of Surgery*.

[21] Wang K., Yang C.-Q., Duan L.-P. (2012). Changing pattern of adenocarcinoma of the esophagogastric junction in recent 10 years: experience at a large tertiary medical center in China. *Tumori*.

[22] Ronellenfitsch U., Schwarzbach M., Hofheinz R. (2013). Perioperative chemo(radio)therapy versus primary surgery for resectable adenocarcinoma of the stomach, gastroesophageal junction, and lower esophagus. *Cochrane Database of Systematic Reviews*.

[23] Hosokawa Y., Kinoshita T., Konishi M. (2012). Clinicopathological features and prognostic factors of adenocarcinoma of the esophagogastric junction according to Siewert classification: experiences at a single institution in Japan. *Annals of Surgical Oncology*.

[24] Xiao W.-G., Ma K., Peng L., Li Q., Chen L.-H., Han Y.-T. (2012). Characteristics of lymphatic metastasis and surgical approach of adenocarcinoma of the esophagogastric junction. *Zhonghua Wei Chang Wai Ke Za Zhi*.

[25] Reeh M., Mina S., Bockhorn M. (2012). Staging and outcome depending on surgical treatment in adenocarcinomas of the oesophagogastric junction. *British Journal of Surgery*.

[26] Curtis N. J., Noble F., Bailey I. S., Kelly J. J., Byrne J. P., Underwood T. J. (2014). The relevance of the Siewert classification in the era of multimodal therapy for adenocarcinoma of the gastro-oesophageal junction. *Journal of Surgical Oncology*.

[27] Kleinberg L. (2013). Therapy for locally advanced adenocarcinoma of the gastroesophageal junction: optimizing outcome. *Seminars in Radiation Oncology*.

